# A Rapid and Low-Cost PCR Thermal Cycler for Low Resource Settings

**DOI:** 10.1371/journal.pone.0131701

**Published:** 2015-07-06

**Authors:** Grace Wong, Isaac Wong, Kamfai Chan, Yicheng Hsieh, Season Wong

**Affiliations:** AI Biosciences, Inc., College Station, Texas, United States of America; Mediterranean Agronomic Institute at Chania, GREECE

## Abstract

**Background:**

Many modern molecular diagnostic assays targeting nucleic acids are typically confined to developed countries or to the national reference laboratories of developing-world countries. The ability to make technologies for the rapid diagnosis of infectious diseases broadly available in a portable, low-cost format would mark a revolutionary step forward in global health. Many molecular assays are also developed based on polymerase chain reactions (PCR), which require thermal cyclers that are relatively heavy (>20 pounds) and need continuous electrical power. The temperature ramping speed of most economical thermal cyclers are relatively slow (2 to 3°C/s) so a polymerase chain reaction can take 1 to 2 hours. Most of all, these thermal cyclers are still too expensive ($2k to $4k) for low-resource setting uses.

**Methodology/Principal Findings:**

In this article, we demonstrate the development of a low-cost and rapid water bath based thermal cycler that does not require active temperature control or continuous power supply during PCR. This unit costs $130 to build using commercial off-the-shelf items. The use of two or three vacuum-insulated stainless-steel Thermos food jars containing heated water (for denaturation and annealing/extension steps) and a layer of oil on top of the water allow for significantly stabilized temperatures for PCR to take place. Using an Arduino-based microcontroller, we automate the “archaic” method of hand-transferring PCR tubes between water baths.

**Conclusions/Significance:**

We demonstrate that this innovative unit can deliver high speed PCR (17 s per PCR cycle) with the potential to go beyond the 1,522 bp long amplicons tested in this study and can amplify from templates down to at least 20 copies per reaction. The unit also accepts regular PCR tubes and glass capillary tubes. The PCR efficiency of our thermal cycler is not different from other commercial thermal cyclers. When combined with a rapid nucleic acid detection approach, the thermos thermal cycler (TTC) can enable on-site molecular diagnostics in low-resource settings.

## Introduction

The ability to make technologies for the rapid diagnosis of infectious disease broadly available in a portable, low-cost format would mark a revolutionary step forward in global public health [1, 2]. A critical challenge to these efforts is that a large segment of the population in need of these advances resides in low-resource settings (LRS) that offer limited laboratory infrastructure [3, 4]. Polymerase chain reaction (PCR) continues to be an indispensable tool in a diverse array of genomic analysis applications. Many molecular assays have been developed around PCR [5, 6], including pathogen and infectious disease detection [7–9], food and water safety [10–12], forensics [13, 14], population-scale polymorphisms [15, 16], and mutation studies [17]. Yet despite advances in PCR technology, it remains largely a laboratory technique, requiring expensive equipment and trained personnel. Thus, PCR-based devices for molecular diagnostics have not been fully commercialized for the geographical areas and demographics that would benefit most from the technology. These areas are typically LRS such as developing countries and rural areas of the United States. Besides the cost and temperature-sensitivity of reagents, thermal cyclers are generally much too expensive to be purchased for users in these areas. In addition, the timescales required to perform a typical PCR remain slow, generally on the order of an hour or more. The large thermal masses of most commercial thermal cyclers make the process very inefficient. To this end, many innovative methods have been reported, aimed at performing PCR faster [18–25]. Although these approaches provide valuable scientific innovation, most of them are difficult to be implemented in LRS.

For example, in well-based PCR, where the reaction is performed in a chamber that is cyclically heated and cooled [26–29], complex control instrumentation is needed to dynamically cycle the temperature of PCR samples [30]. Also, the thermal mass associated with the heater and PCR well limits the achievable thermal ramp rates, increasing reaction times [26]. In shuttle PCR, where thermal cycling is performed by shuttling small plugs of the PCR mixture back and forth between isolated temperature zones [31, 32], the limitations set by the thermal mass of the system are eliminated by designing the temperature variations to occur spatially, instead of over time. Although shuttle PCR removes the challenges of dynamic temperature cycling, shuttle PCR introduces technical challenges with respect to fluid handling [22, 26] since the transport of the PCR mixture becomes the time-dependent feature of the device. Continuous-flow PCR (CF-PCR) is performed by pumping the PCR mixture at a steady volume flow rate through a microfluidic channel, passing it through different temperature regions [32–37]. The temperature cycling is achieved by fabricating a single serpentine channel that passes repeatedly through distinct temperature regions of the microfluidic chip. Because the liquid must travel a long distance in the serpentine channel, there can be significant reagent adsorption to the surface of the channel. There is also a report of using the heat-sink of a desk-top computer to drive PCR’s three distinct steps by placing PCR tubes in the heat-sink; but the speed of the reaction is slow (needing 5 min to complete one cycle) [38]. Recently, extremely fast PCR protocols (0.4 to 2.0 s per cycle) were reported using 10- to 20-fold primers and polymerase concentration to drive the reaction targeting relatively short targets [25]. However, because of the high concentrations of polymerase and primers used, reactions had to be prepared on ice, quickly transferred to the PCR thermal instrument, and immediately amplified without delay, thus making the setup very difficult to implement in LRS [25].

As a result, there remains an unmet need for a rapid and low-cost thermal cycler that can carry out molecular diagnostics in rural areas and developing countries in a rapid and low-cost manner. In this work, we describe a practical solution for bringing a low-cost, simple-to-operate, and rapid thermal cycler to under-served and developing populations. We demonstrate fast PCR thermal cycling at a rate of 15 to 30 s per cycle using a device that can be assembled for $130 using commercial off-the-shelf (COTS) items.

This so-called thermos thermal cycler (TTC) uses a very simple design to perform PCR amplification which is based on the "archaic" method of hand-transferring reaction tubes through a series of water baths that would minimize the temperature ramping time needed for PCR tubes to reach thermal equilibrium. These traditional water baths all need active thermal control, so they are not suitable for use in LRS.

**Fig 1:**
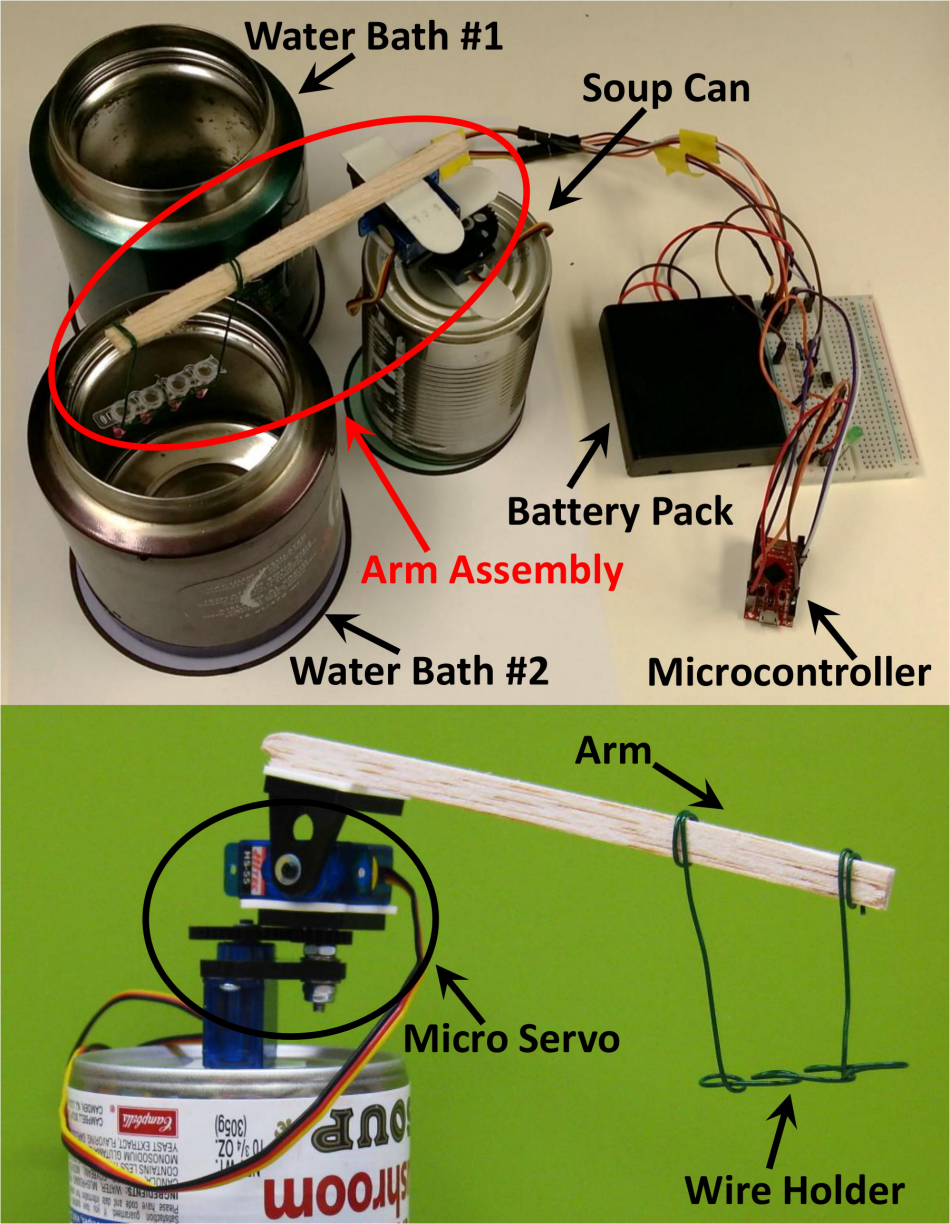
Setup of the low-cost and rapid TTC. Major components include two 16 oz thermoses, a pan-and-tilt servo set to control the up and down and rotational motion to shuttle PCR vessels in and out of the thermoses. Also included are the battery pack, the Arduino electronic controller, and a breadboard. To reduce cost, the pan-and-tilt setup is constructed using a soup can, a wood stick, and a PCR tube holder made by metal wire. The parts were glued together using removable tape.

A key element of the device architecture in our new approach is that it uses Thermos stainless steel vacuum-insulated food jars (to store the water that heats the PCR tubes) and a layer of cooking or mineral oil on top of the water to maintain a stable temperature in the 20 to 30 minute time span that is needed to complete the PCR reaction in a TTC (Fig 1). First, water has a high specific heat capacity, which greatly reduces temperature fluctuations when compared to approaches of using heated air in a thermal cycler (e.g., the LightCycler or the Rotor-Gene Q cycler by Roche Life Science and Qiagen, respectively); and a boiling point very near the DNA denaturation temperature, which makes it a convenient denaturation source requiring negligible control for overheating (only steam will be produced). Water is also readily available and can be added into the TTC only when needed, thus reducing the weight of the device. The oil is the key component which prevents significant heat loss from the surface of the water so that the water in the thermos that performs denaturation has a sufficiently high temperature throughout the cycling duration to carry out the nucleic acid denaturation. Because we are able to maintain sufficient temperatures without active heating or cooling once the water is heated, a stable and continuous power supply is no longer needed during the PCR reactions. To perform two or three-step PCR, two or three thermoses, maintained at denaturation and annealing/extension temperatures, are needed.

During the experiments, the thermos for DNA denaturation can maintain its temperature between 98 and 93°C. At this temperature range, DNA can be rapidly denatured. Melting curve analyses of PCR amplicons have demonstrated that most DNA or PCR amplicons melt/denature completely at 90°C and below [39], therefore, the slight temperature drop throughout the duration of the PCR reaction will not prohibit denaturation and the subsequent annealing of primers to the denatured DNA.

The second thermos can aid primer annealing and target extension (55–65°C for the samples used in the experiment). The temperature drop in the thermos for annealing and/or extension is minimal in our set up as the thermal difference between the water inside and the ambient environment is much smaller than the thermos used for DNA denaturation. The automation of the PCR reactions by an Arduino microcontroller is performed so that the PCR tubes are mechanically transferred by the actions of servo motors to carry out the necessary PCR steps as described in the next section.

For assays that use separate annealing and extension steps, a third water bath (e.g., at 72°C) may be included in PCR reactions. Traditionally, the third step is used to speed up the target extension process as the polymerase tends to be more active at high temperatures; but most modern PCR reactions (e.g., TaqMan assays) use only two temperatures to yield amplicons (e.g., at 95°C and 60°C) because the modern day enzymes used are generally faster.

In addition to being able to maintain temperatures in a low-cost manner, water (in comparison to air) provides very speedy heat transfer to the reagents inside a PCR vessel so the right temperature can be quickly reached, thus reducing the time needed to perform PCR. This allows the reactions to be performed at a very high speed while keeping the cost low enough to be used in LRS. Others have tried improving the speed of the PCR reactions by engineering different approaches (forcing the exchange of hot and cold water to the reaction tubes) or reducing the reaction volume; but the cost and difficulty associated with these approaches are generally much higher than the design presented here [40, 41].

Furthermore, while a thermal block requires the use of matching PCR tubes to ensure efficient heat transfer between the block and the tubes, our water-based TTC can accommodate all industry-standard PCR tubes, including 0.1 to 0.5 mL PCR tubes, plastic/glass capillary tubes, as well as arrayed thin-film (Arrayed Tape by Douglas Scientifics), which houses many PCR reactors with volumes as low as 0.8 μL (data not shown). This flexibility makes it amiable to conventional protocols and peripheral hardware.

## Materials and Methods

### Setting up water baths for TTC

For our typical reactions involving two-step PCR, two 16 oz Thermoses (Thermos, catalog number NS340TL4, purchased from a local Target store) were used with each thermos being prepared with water and oil at denaturation (95°C) or annealing/extension temperatures (55, 60, 63, or 65°C). A third Thermos (72°C) was also set up when 3-step PCR was necessary.

We first used a household electric kettle to boil water to 100°C. To prepare the thermos designated for denaturation, boiling water was immediately added to the thermos that was already filled with 11 mL of cooking or mineral oil. The boiling water would heat up the thermos and the oil, and its temperature would then drop a few degrees because of heat loss. The exact temperature of this hot bath is not critical as long as it starts over 95°C and stays over 90°C during the PCR reaction.

To achieve the right temperature (e.g. 55, 60, 63, 65, or 72°C) for primer annealing and/or target extension by polymerase, we first filled the thermos with tap water (equilibrated to room temperature at ~25°C) and 11 mL of cooking or mineral oil, then added boiling water to the level where the thermos’ double-wall ends. The exact volumes of tap water and boiling water needed were determined empirically according to the desired final temperature of the water bath (S1 Table), and roughly based on the estimates provided by the following formula:

$$m_{h}\times \left(4.18\;J/g{}^{\circ}C\right)\times \left(100{}^{\circ}C-T\right)=m_{c}\times \left(4.18\;J/g{}^{\circ}C\right)\times \left(T-25{}^{\circ}C\right)$$

where 4.18 J/g°C is the specific heat of water, m_h_ is the mass of the boiling water at 100°C, m_c_ is the mass of the tap water at 25°C, and T is the final temperature of the water bath.

### Automation of TTC

Automation of the TTC was achieved by programming a servo motor assembly to shuttle PCR vessels between the water baths (Fig 1). A “sub-micro pan & tilt” assembly (model 3: SPT500) driven by two micro servos (HS-55, Servocity, Winfield, KS) were controlled by an Arduino-compatible microcontroller (Pro Micro 5V/16MHz, Sparkfun, Boulder, CO). Precise panning movements by the pan micro servo shuttled the sample between the different water baths in a circular path. Controlled up-and-down movements were provided by the tilt micro servo that moved PCR vessels in and out of the thermoses. An arm assembly made of a small wood stick (which can be replaced by plastic or aluminum plates if desired) extended the reach of the servos. The entire arm assembly was simply mounted onto a soup can with removable tape (Command removable strips, 3M). Thin metal wire was used to make a holder for the PCR vessels (plastic tubes and glass capillary tubes). A 6V battery pack (4 AA batteries) powered the servos and controller.

Servos are an attractive option because of their low cost, robustness and wide variety of compact sizes and capacities. The servo controller provides quick, intuitive access for motion control programming by a user. When connected to a PCR or laptop computer, the user interface allows real-time access to the system and on-the-fly servo control. When disconnected from the user interface, automated programs can also run the controller from its memory at start up, allowing computer-free, independent operation. The retail cost for the materials, including the two thermoses, is estimated to be $130. Major components used in building the TTC are listed in Table 1.

**Table 1:** Major items needed to setup the TTC to perform rapid PCR amplifications.

Part	Quantity	Cost
16 oz Thermoses (Target, NS340TL4)	2	$42.00
Arduino uno (Radioshack, 276–128)	1	$29.99
400 point breadboard (Pololu, 351)	1	$3.75
M-M jumper wires (Pololu, 1702)	14	$3.50
Mini pushbutton switch (Pololu, 1400)	1	$0.19
4-AA battery holder, enclosed with switch (Pololu, 1159)	1	$1.75
4 pack AA batteries (Radioshack, 2300849)	4	$5.00
HS55 Servos (Servocity, 31055S00)	2	$19.98
Direct drive pan and tilt kit (Servocity, SPT100H)	1	$19.99
Various Miscellaneous Parts (soup can, tape, etc.)		$3.00
**Total Cost**		**$129.15**

### Use of glass capillary tubes for PCR

Unlike traditional aluminum block thermal cyclers that need compatible brands of PCR tubes for effective heat transfer, water baths accept PCR vessels of almost any kind. The vessels can be simply partially-immersed into the water baths to carry out the reaction steps. The reaction will go well as long as the reagents are placed below the oil level. We used the Roche LightCycler glass capillaries (20 μL capacity) (Roche Diagnostics, Indianapolis, IN) to perform very rapid PCR reactions due to the faster heat transfer one can get over polypropylene PCR tubes [42, 43]. The high-aspect ratio geometry of the glass capillary tubes also aided the water bath to heat and cool the reagents faster.

### Temperature measurement setup

To compare the TTC’s performance to a commercial thermal cycler, we placed a 0.2-mL PCR tube and glass capillary tube containing deionized water (20 μL and 17 μL, respectively) and a thermocouple (TC) into the TTC sample holder and measured the temperature inside the water during thermal cycling. The temperature was measured using K-type thermocouples (Model No. SC-TT-K-30-36, Omega Engineering, Stamford, CT) connected to a data logger thermometer (HH147U, Omega Engineering, Stamford, CT). To create a temperature probe assembly, the PCR tube cap was punctured using a 20-gauge syringe needle; the thermocouple was then inserted through the small hole and secured on the cap by using a glue gun to deposit melted glue gun materials. The same type of TCs was also used to record the temperature of the water baths by directly immersing the TCs into the water inside the thermoses.

### Total run time definition

In traditional single-thermal block commercial thermal cyclers, the total run time is calculated based on the hot-start duration, the dwell time specified in the denaturation / annealing / extension steps, as well as the ramping time between temperature set points (usually takes 10 to 20 s). With the TTC, we calculate the total reaction time by counting the initial hot-start time, the time needed to transport the tubes between the thermos (about 0.79 s per movement) and the time that the tubes stayed in the water bath. For example, in a TTC protocol, a 40-cycle reaction that includes 2 min of hot-start step, 7 s of denaturation and 8 s of annealing/extension steps will only need 13 min and 3 s to complete (2 min hot-start, 10 min incubation in water-baths, plus 63 s for shuttling the tubes).

### Gel electrophoresis analysis

After amplification by the TTC or the commercial unit, the PCR amplicons were typically evaluated using a 1.2 or 2.2% pre-casted gel (Flash Gel by Lonza, Rockland, ME) at 275 volts for 7 minutes (4 μL mix added to 1 μL of loading dye). The resulting gel images were captured using a cellphone camera (Note 3 Smart Phone, Samsung Electronics). The typical ladder sizes used in the gel were 50, 100, 150, 200, 300, 500, 800, 1500 bp.

### PCR conditions

We conducted numerous reactions in different conditions throughout the investigation to demonstrate that the TTC is fast, efficient and compatible with a range of targets from 139 to over 1,500 bp. The work reported here will demonstrate that the TTC could produce target amplicons in a much shorter amount of time using the same number of cycles as a commercial thermal cycler. All commercial thermal-block-based PCR runs were performed in a CFX96 Touch real-time thermal cycler (Bio-Rad, Hercules, CA). Polypropylene plastic tubes and glass capillary tubes were also used to demonstrate its flexibility in terms of accepting various tubes to deliver fast heat transfer in water. While there is no heated-lid in our design, we did not have to use silicone oil layer over the PCR mixtures in polypropylene PCR tubes to minimize evaporation. This is because the time that the polypropylene tubes spent at elevated temperatures in a TTC is much shorter than when the protocol was carried out in traditional thermal block cyclers (hence more rapid cycling). As a result, only minimal evaporation and condensation was observed and that did not affect the quality of the PCR amplification in an observable manner (such as by gel electrophoresis).

Table 2 summarizes the targets, primer sequences, product sizes and the components of the PCR reactions. The PCR protocols will be described in the corresponding data sections. Stock primer concentrations were 10 μM and were diluted to a final concentration of 500 nM after the master mixes were prepared. Unless specified otherwise, reaction volumes for the commercial cycler and TTC were 20 μL. The sample volume for glass capillary tubes was 17μL.

**Table 2:** Target, primer sequences, target sizes and components of PCR mix.

Target (PCR amplicon sizes)	PCR components
*Bacillis subtilis* (139 bp)	10 μL 2x SsoAdvanced SYBR-Green supermix
	1 μL For primer (5’ TGT AAG CCA TAA GCC ATT CG 3’)
	1 μL Rev primer (5’ GCT ATC ATC CCA ATC TCC GA 3’)
	3 μL nuclease-free water
	5 μL template
	Total volume 20 μL
Human DNA (600 bp, 500 bp, 400 bp, 300 bp, 200 bp, 100 bp)	7.5 μL Isohelix DNA Quality Check Kit human primer mix
	12.5 μL Isohelix DNA Quality Check Kit amplification mix
	2.5 μL nuclease-free water
	2.5 μL human genomic DNA
	Total volume 25 μL
*Neisseria gonorrhoeae porA* pseudogene (142 bp)	10 μL 2x SsoAdvanced SYBR-Green supermix
	1 uL For primer (5’ TGG AGC ATG TGG TTT AAT TCG A 3’)
	1 uL Rev primer (5’ TGC GGG ACT TAA CCC AAC A 3’)
	5 uL nuclease-free water
	3 uL template
	Total volume 20 μL
16S rRNA gene (~1,522 bp)	10 μL 2x PrimeSTAR Max mix
	1 μL For primer (5’ AGA GTT TGA TCC TGG CTC AG 3’)
	1 μL Rev primer (5’ ACG GCT ACC TTG TTA CGA CTT 3’)
	4 μL nuclease-free water
	4 μL template (at various concentrations)
	Total volume 20 μL
*Staphylococcus aureus nuc* gene (281 bp)	10 μL 2x PrimeSTAR Max mix
	1 μL For primer (5’ GCG ATT GAT GGT GAT ACG GTT 3’)
	1 μL Rev primer (5’ AGC CAA GCC TTG ACG AAC TAA AGC 3’)
	6 μL nuclease-free water
	2 μL template
	Total volume 20 μL

#### *Bacillis subtilis* target

Primer pairs targeting a 139 bp region of *B*. *subtilis* genomic DNA (ATCC 31785) were used in PCR reactions using TTC and a commercial cycler. Genomic DNA was extracted in house and its stock concentration is estimated to be (10^3^ copies/μL). Primers and template were mixed with SsoAdvanced SYBR Green Supermix (Bio-Rad, Hercules, CA) and placed in 0.2 mL low-profile PCR tubes (Bio-Rad Laboratories, Hercules, CA). The commercial PCR conditions were as follows: 2 min of hot-start at 95°C, followed by 40 cycles of [95°C (10 s) and 60°C (35 s)]–with a total reaction time of ~58 min. The TTC PCR condition was almost identical to the commercial runs: 2 min hot-start at 95°C, followed by 40 cycles of [95°C water bath (10 s) and 60°C water bath (35 s)]. After the reactions, the tubes were placed on the top surface of a blue LED gel illumination box (Lonza, Walkerville, MD). The SYBR Green fluorescent signal from the tubes was captured by a cell phone camera (Note 3, Samsung Electronics) with an orange filter placed in front of the phone camera’s lens to capture the green fluorescence.

#### Multiplexed PCR reactions

To demonstrate its ability to carry out multiplexed reactions, a commercially available multiplexed PCR kit (Isohelix DNA Quality Check (DQC) Kit) was purchased and the positive control human DNA template was used in PCR reactions. The Isohelix kit typically yields multiple proprietary amplicons at 100, 200, 300, 400, 500 and 600 bp. The 500-bp fragment is derived from an internal control and is present even in no-template controls. Commercial PCR reactions were performed in the following condition: 5 min hot-start at 95°C, followed by 30 cycles of [95°C (30 s), 63°C (30 s), and 72°C (45 s)]. TTC-based reactions were performed using three water baths because of the three temperature protocol recommended in the kit. We took an empirical approach to shorten the incubation time in each water bath: 3 min hot-start at 95°C, followed by 40 cycles of [95°C (7 s), 63°C (10 s), and 72°C (10 s)].

#### PCR amplification using clinical samples

To demonstrate the practical value of the TTC, we used it to amplify genomic bacterial DNA extracted from clinical samples of previously collected urine samples found to be positive with *Neisseria gonorrhoeae*. PCR reactions targeting 132 bp *N*. *gonorrhoeae porA* pseudogene using the SsoAdvanced SYBR Green Supermix was performed. Commercial PCR reactions were performed with the following condition: 2 min hot-start at 95°C, followed by 40 cycles of [95°C (10 s) and 55°C (45 s)]. This took 67 min to complete 40 cycles. The TTC PCR was performed with the following condition: 2 min hot-start at 95°C, followed by 40 cycles of [95°C (10 s) and 55°C (20 s)].

#### Amplification of the 16S rRNA gene

A 16S rRNA ReadyMade primer set (Integrated DNA Technologies, Coralville, Iowa) that yields a ~1,522 bp product was used to demonstrate the rapid amplification of long PCR targets using the TTC. The template was DNA extracted from *N*. *Gonorrhoeae* and prepared in PrimeSTAR Max DNA Polymerase master mix (Takara/Clontech Laboratories, Inc., Mountain View, CA). Commercial reactions (including no template controls) were performed with the following condition: 2 min hot-start at 94°C, followed by 40 cycles of [98°C (10 s) and 65°C (40 s)]–with a total reaction time ~60 min. Two-step PCR using both plastic and glass tubes was performed in the TTC with various conditions to optimize the incubation time without compromising amplification efficiency: 90 s hot-start at 95°C, followed by 40 cycles of [95°C (9 or 12 s) and 65°C (20 or 30 s)].

#### Demonstrate the speed and sensitivity of reaction by the TTC

After demonstrating that the TTC can amplify a wide range of target sizes, we focused on improving the speed and sensitivity limits of the TTC set up in using regular polypropylene plastic tubes. By using extracted *Staphylococcus aureus* (ATCC 29213) template, PCR reactions targeting the *nuc* gene (281 bp) were performed using PrimeSTAR Max DNA polymerase master mix in thin-walled PCR tubes (Cat. No. 16950, Sorenson Bioscience Corp, Murray, UT) to amplify the extracted template [44]. The reaction mixes were made with serially diluted template to allow us to prepare each reaction using template that contained approximately 1, 10, 100 or 1000 copies/μL of *S*. *aureus* genome copies. The commercial PCR reaction was 2 min of hot-start at 95°C, followed by 40 cycles of [95°C (10 s) and 60°C (20 s)] for a total of 48 min and 18 s. TTC condition for reactions in plastic tubes was 2 min of hot-start at 95°C, followed by 40 cycles of [95°C (11 s) and 60°C (17 s)]. TTC reaction condition in glass capillary tubes was 2 min of hot-start at 95°C, followed by 40 cycles of [95°C (7 s) and 60°C (8 s)].

## Results and Discussion

### Using oil to maintain water temperature

**Fig 2:**
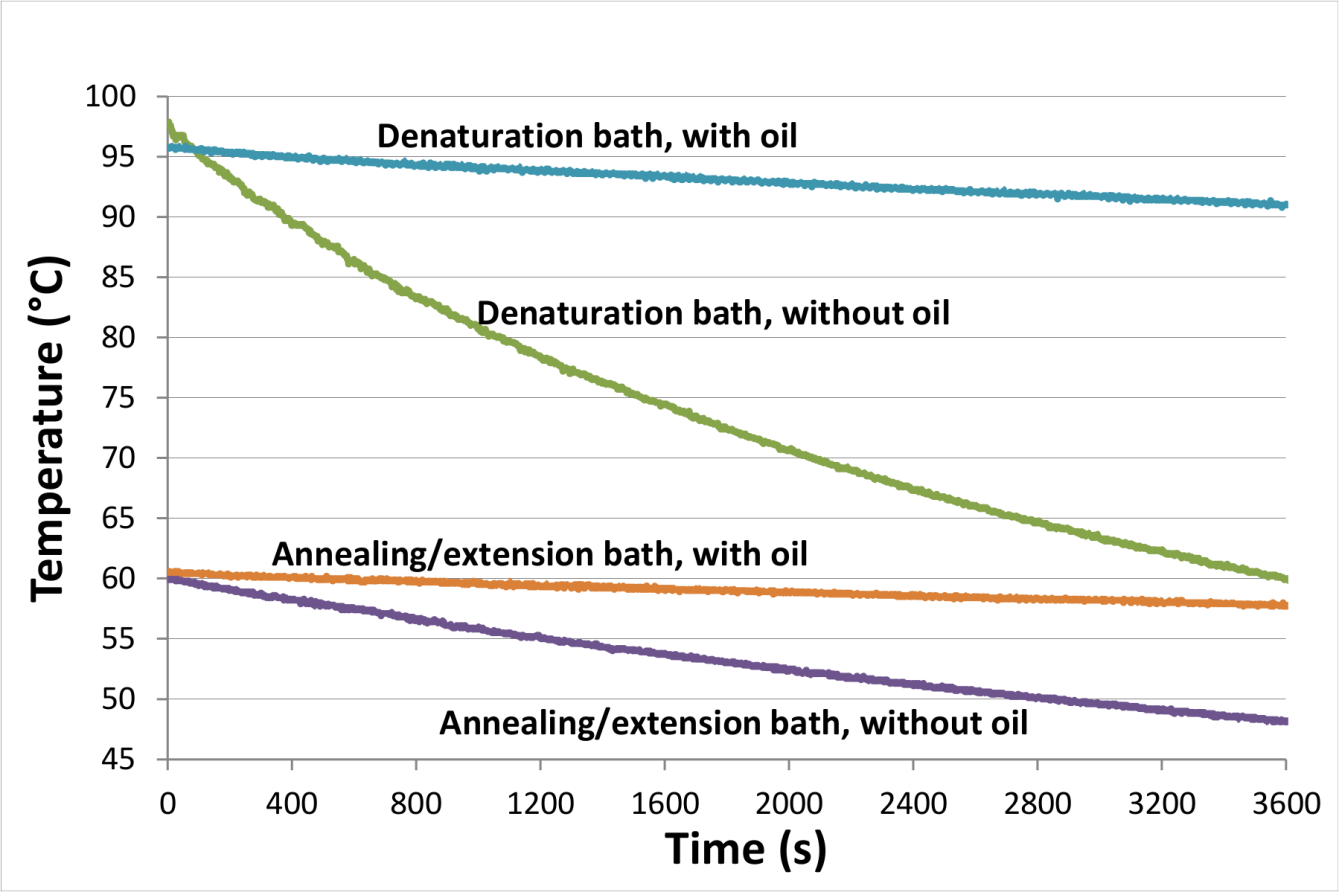
Effect of using oil as an insulation to keep water above 90°C for denaturation step during PCR. The water temperature inside the thermos drops significantly faster if oil is not used to insulate the water. Oil can maintain the denaturation and annealing/extension baths’ water temperature very well for over 1 hr. Because our typical 40-cycle reactions does not last longer than 30 min; PCR efficiency carried out by the TTC does not drop significantly even near the end of reaction when exponential growth is having its most effect.

We used vacuum-insulated stainless steel food-jars and an oil layer to achieve a low-cost method to passively, but effectively, maintain the temperature of the water baths for PCR. These COTS Thermos food-jars can maintain water temperature effectively only if their caps are tightly closed to prevent heat loss. To perform water bath-based PCR, the caps have to be removed to allow partial immersion of the tubes which will lead to a rather fast temperature drop during the reaction if only water is used. By adding a layer of oil on top of the heated water, we were able to maintain the water temperature in the thermos extremely well (over 90°C for 1 hr) for hot-starting the polymerase and denaturing the target DNA (Fig 2). In our 16-oz thermos (~473 mL max. capacity), as little as 11 mL of oil can sufficiently cover the water surface and build a layer of insulation. Starting at an initial temperature of 95.7°C, the temperature dropped only 4.7°C to 91.0°C in 60 min. Without the oil, the water temperature dropped over 37°C in the same time span. For the annealing and extension steps, which usually take place between 50 and 75°C, the oil and the thermoses help to maintain the temperature bath even better because of the lower temperature difference between the ambient environment and the water inside the annealing/extension bath. For example, in Fig 2 the temperature dropped only 2.7°C in an hour (from 60.5 to 57.8°C) when the oil layer was present. However, the temperature dropped 11.8°C (from 60.0 to 48.2°C) without the oil layer. It is important to note that since we carry out our standard 40-cycler reactions in the 13 to 30 min. time frame, the temperature drop of less than 2.7°C within the denaturation thermos is insignificant. Our data (not shown) also indicates that both cooking oil and mineral oil provide very effective insulation and the difference is negligible, so the use of cooking oil is recommended due to its lower price.

### Thermal ramping speed

Rapid heating and cooling of the reagent mix is critical for reducing the time needed for each PCR cycle. The TTC format eliminates the heating and cooling of the thermal block but the reagents still need to be heated and cooled once they enter the water baths. Table 3 summarizes the measured ramping rates of the reagents between 95 and 60°C under different PCR setups. We measured how fast the water heats and cools inside the vessels placed in the TTC and two commercial thermal cyclers (Bio-Rad CFX96 real-time thermal cycler and Eppendorf Mini cycler). Thermal probes were immersed in 20 μL and 17 μL of water inside the plastic tubes and glass capillary tubes, respectively.

**Table 3:** Ramping speed of the reagents measured with a thermocouple placed inside of a PCR vessel filled with water (between 60 and 95°C).

	Heating (°C/s)	Cooling (°C/s)
Eppendorf Mastercycler personal	0.6	0.5
Bio-Rad CFX96 real-time system	1.2	1.1
Bio-Rad plastic PCR tubes in TTC	5.0	6.0
Roche Glass capillary tubes in TTC	15	13

Measured inside 0.2-mL tubes or glass capillary tubes filled with 20 μL or 17 μL of water, respectively.

The information in the Table 3 shows that the measured temperature ramping speed of water in thermoelectric-based thermal block cyclers was 1.2°C/s or less. For TTC, water reached denaturation (95°C) and annealing/extension temperatures (60°C) very quickly; with regular PCR tubes having heating/cooling rates of 5°C/s and 6°C/s, respectively. With a higher heat conductivity and larger aspect-ratio (height/diameter) than plastic, glass capillary tubes provide the fastest heating/cooling rates of 15°C/s and 13°C/s, respectively. The results from temperature ramping measurements indicate that we should be able to perform PCR very rapidly using our simple setup.

**Fig 3:**
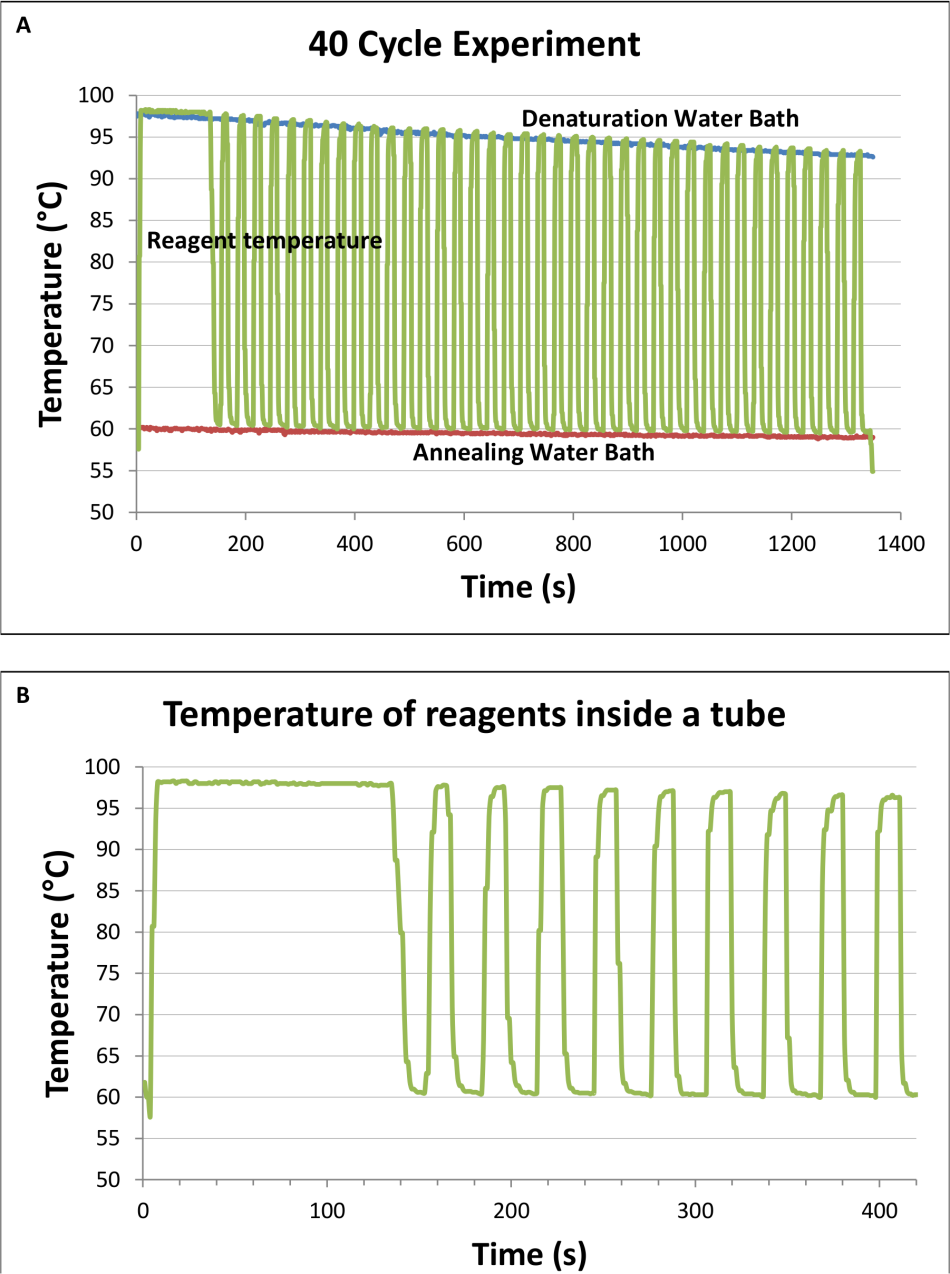
Temperature plot of reagent temperature inside a glass capillary tube measured using a thermocouple. **(A)** The plot recorded 2 min of hot-start step, followed by 40 cycles of moving the tubes between the denaturation (10 s) and annealing/extension (20 s) water baths. The temperature ripple observed is insignificant. **(B)** The expanded view of the temperature plot that covers the hot-start step and the first 10 cycles. A rough measurement shows that the material inside the capillary tube can reach denaturing temperature in 6 s, and annealing/extension temperature in 10 s.

Fig 3 shows the temperature readings from the thermal probe inside a water-filled glass capillary tube during a 40-cycle PCR run. The plot recorded the 2 min hot-start process and the incubation of the tubes in the denaturation water bath for 7 s and annealing/extension water bath for 13 s. The water temperature in the two baths was also monitored throughout the reaction. The temperature ripple observed is insignificant. The expanded view from the hot-start and the first 10 PCR cycles were also included.

We wish to emphasize that because we chose not to use active temperature controls, our TTC setup cannot fully maintain a fixed temperature as a small amount of heat will always be lost to the ambient environment continuously. However, although a slight temperature drop will occur during the PCR (more drastically in the denaturation water bath), and continue to do so towards the end of the reaction, the temperature drop does not appear to have adversely affected the PCR reactions tested. This is because towards the end of a PCR run, the amplicons will be mostly amplified from the target amplicons generated earlier in the reaction and not from the genomic DNA itself. Typical melting temperature of most PCR amplicons is well below 90°C. This means denaturation of DNA can occur at a lower temperature and faster than at the beginning of the PCR run when mostly genomic targets are denatured (e.g., first 5 to 10 cycles).

### PCR amplification using TTC

#### *Bacillis subtilis* target

**Fig 4:**
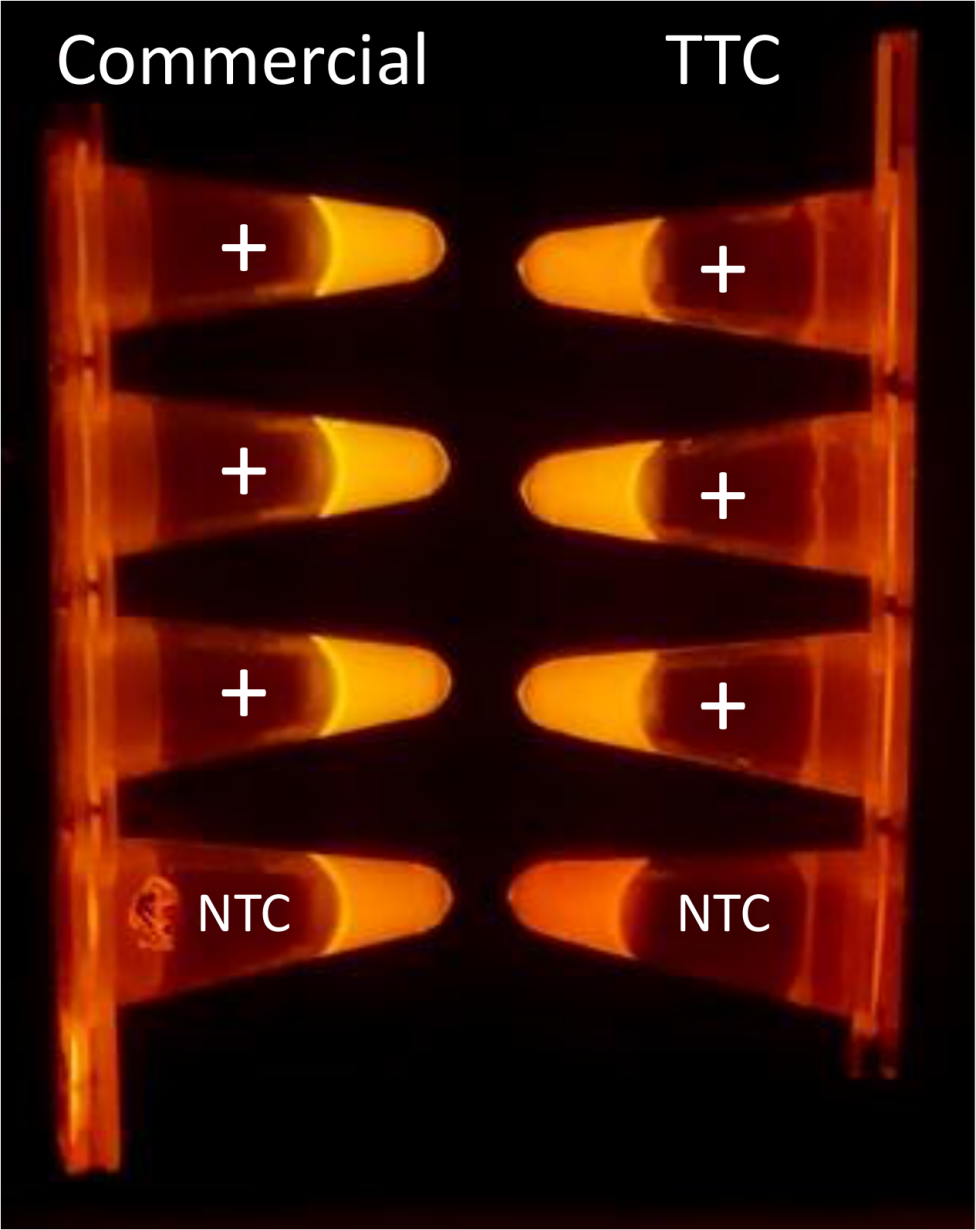
Photos of the PCR tubes illuminated with blue LED. Fluorescent photos of SYBR-Green based PCR reagents tubes after amplification steps in a commercial cycler and the TTC. The intensity of NTC tube is much lower than the tubes containing template DNA.

We first used the TTC to amplify a 139 bp region of gram-positive *B*. *subtilis* genomic DNA. Four tubes were placed in a commercial thermal cycler and an identical set was used with the TTC. In each set, one tube is a no-template control (NTC) while the three other tubes contain an identical amount of *B*. *subtilis* DNA templates extracted in our laboratory (~1,000 copies/μL). Fig 4 is the photo of the PCR tubes after the reaction which clearly shows that the three samples with extracted *B*. *subtilis* template DNA have higher fluorescent signals than the NTC’s. While the commercial thermal cycler protocol took 58 min to complete, the TTC took only 30 min to complete a 40-cycle reaction, resulting in 28 min of time savings.

#### Multiplexed PCR reactions

**Fig 5:**
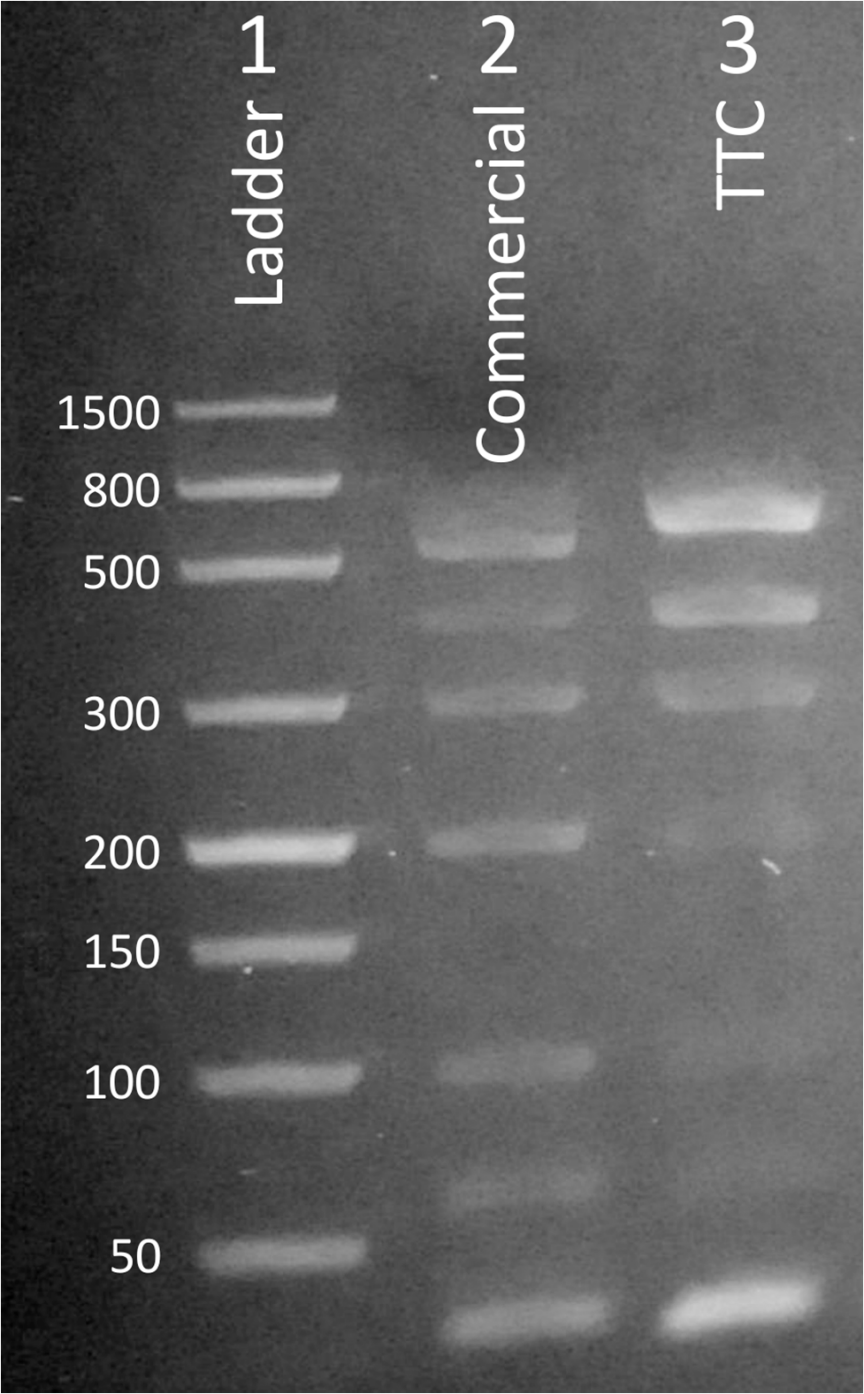
Multiplexed PCR reactions performed with TTC. Lane 1: Ladder: 50/100/150/200/300/500/800/1500 bp. Lane 2: amplicons produced by the commercial thermal cycler (30 cycles in 75 min). Lane 3: amplicons produced by the TTC (40 cycles in 28 min).

Using the Isohelix DQC kit, we ran the PCR using three thermoses and used gel electrophoresis to confirm that multiple targets could be amplified. The gel photo in Fig 5 shows that the TTC can produce multiplexed amplicons with the correct sizes. While additional cycles were needed to produce a similar amount of amplicons (as evident by gel band intensity), the 28 min reaction time needed by the TTC to perform 40 cycles is much shorter than the protocol performed with commercial thermal cycler (75 min for 30 cycles). We are confident that with some reaction optimization, such as using a longer incubation time at annealing/extension temperatures, can make each PCR cycle as efficient as those achieved by commercial units.

#### PCR amplification from clinical samples

**Fig 6:**
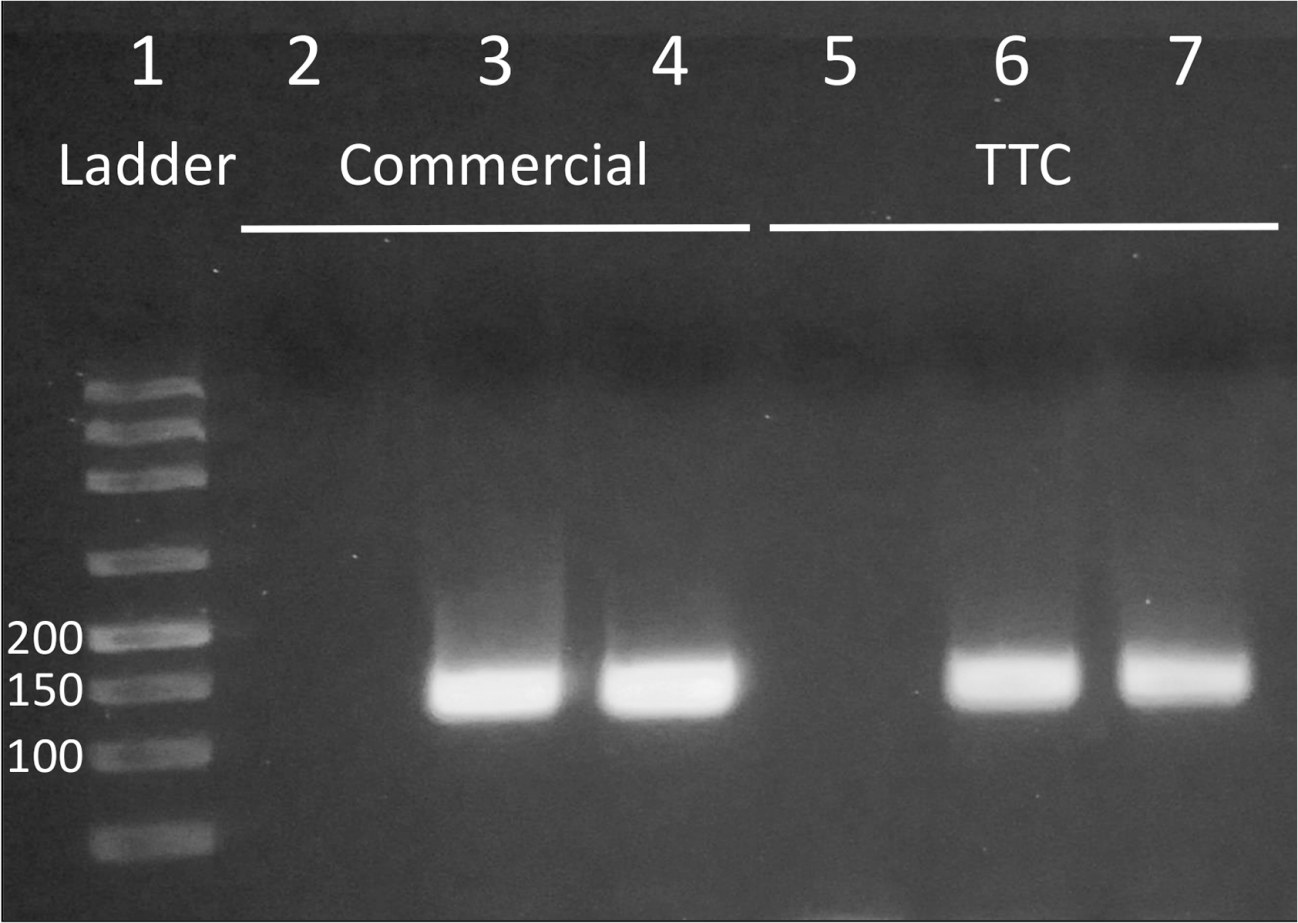
PCR amplification of *Neisseria gonorrhoeae porA* pseudogene from clinical samples. Lane 1: ladder 50/100/150/200/300/500/800/1500 bp. Lanes 2 to 4: NTC and two positive samples performed with commercial cycler. Lane 5 to 7: NTC and two positive samples performed with TTC.

To demonstrate its practical value, we used the TTC to amplify genomic DNA extracted from clinical samples. PCR reactions targeting 132 bp *Neisseria gonorrhoeae porA* pseudogene using the SsoAdvanced SYBR Green Supermix (Bio-Rad) were tested. The gel electrophoresis data from commercial and TTC reactions are shown in Fig 6. TTC was able to amplify clinical sample-derived bacterial DNA rapidly in 25 min while the commercial protocol took 67 min.

#### Amplification of the 16S rRNA gene

**Fig 7:**
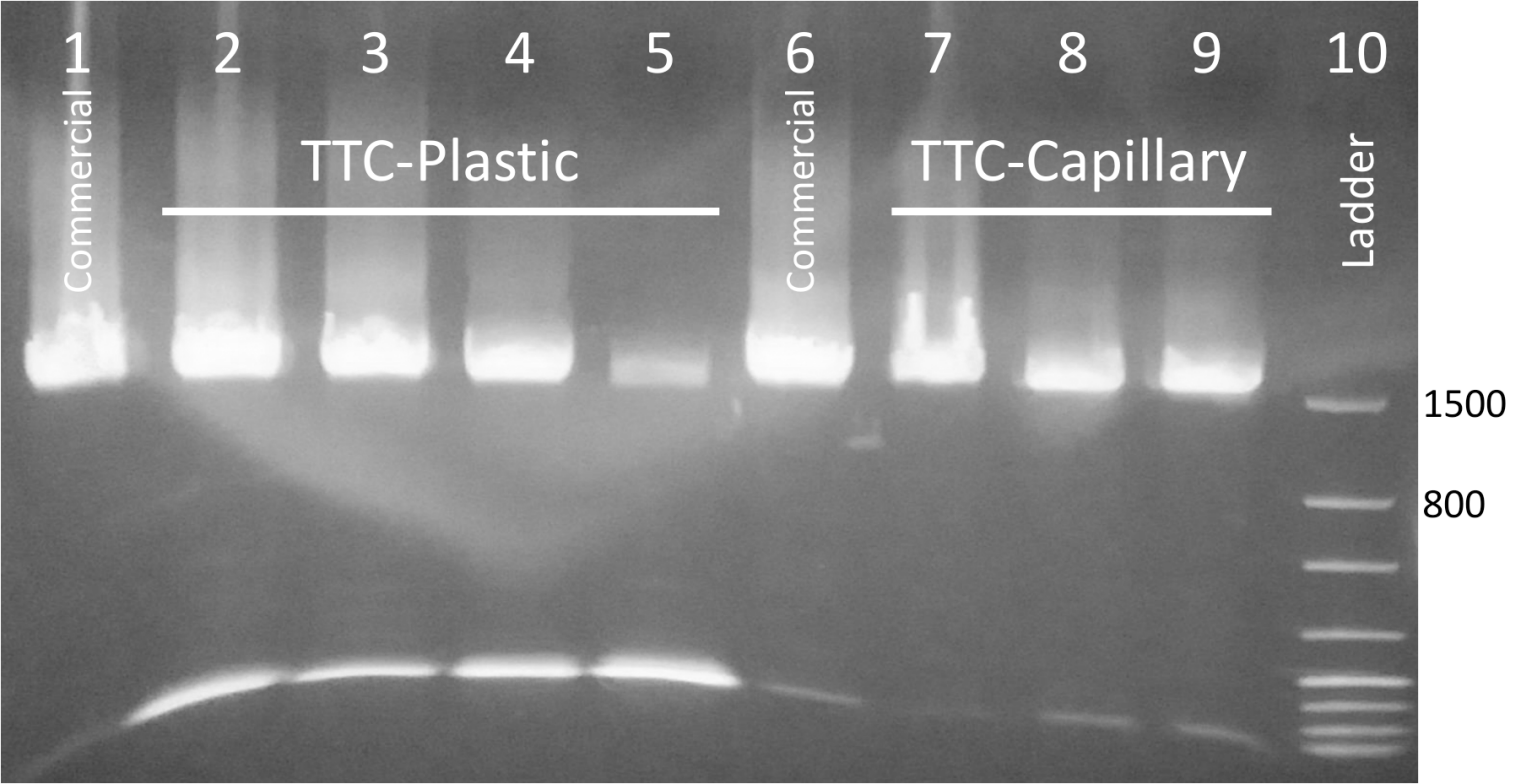
TTC is able to amplify targets over 1,500 bp long in less than 23 min. Lanes 1 and 6 are amplicons from the commercial run. Lane 2 is amplicon from a TTC run of 12 s / 30 s. Lane 3 is from a run of 12 s / 20 s. Lane 4 is a run of 9 s / 20 s (23 min); Lane 5 is also a run of 9 s / 20 s, but some water got in during the run. Lanes 7 to 9 are amplicons collected from glass capillary tubes with the following protocols 12 s / 30 s, 12 s / 20 s, and 9 s / 20 s, respectively. Lane 10 is ladder: 50/100/150/200/300/500/800/1500 bp.

Using primers to target the entire 16S rRNA gene of bacteria, the TTC produced similar amounts of target (~1,522 bp) when compared to the commercial cycler and the results were confirmed by gel electrophoresis (Fig 7). Initially, a longer incubation time at the annealing/extension step by TTC was chosen prior to optimization. Lane 1 and 6 are amplicons generated from the commercial thermal cycler (60 min). Lane 2 is amplicon from a TTC reaction with 12 s (denaturation) and 30 s (annealing/extension) steps (total time of 31.1 min). Lane 3 is amplicon from a TTC reaction with 12 s (denaturation) and 20 s (annealing/extension) steps (total time of 24.4 min). Lanes 4 and 5 are TTC reactions with 9 s (denaturation) and 20 s (annealing/extension) steps (total time of 22.4 min). Note that lane 5 produced a weaker band because the tube’s cap popped out during the run and some water/oil got into this particular PCR tube. Lanes 7 to 9 are amplicons produced in glass capillary tubes using the three conditions and reagents as the plastic tubes. Our result demonstrates that using a TTC, a 40-cycle reaction targeting a ~1,522 bp segment of bacterial DNA can be completed in as little as 22.4 min using both plastic and glass capillary tubes.

### Speed and sensitivity demonstration

**Fig 8:**
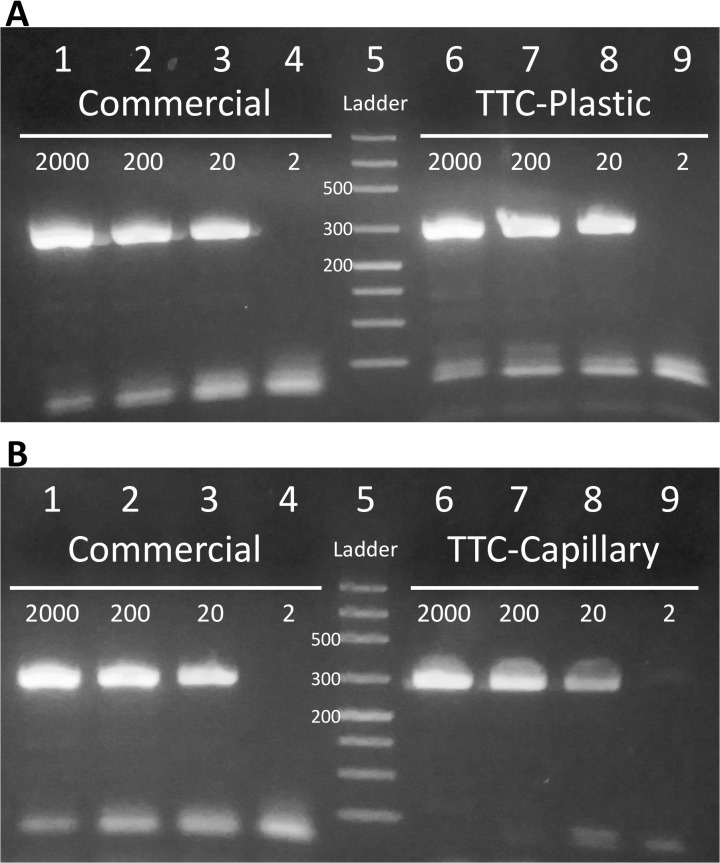
Speed and sensitivity of PCR reactions demonstrated by TTC. PCR reactions to amplify 281 bp of the *nuc* gene from 2000, 200, 20, or 2 copies of *S*. *aureus* genomic DNA. **(A)** Lanes 1 to 4: PCR products from commercial thermal cycler. Lane 5: ladder. Lanes 6 to 9: PCR products from TTC with thin-walled plastic tubes, using a protocol of 2 min hot-start, followed by 40 cycles of 11 s and 17 s denaturation and annealing/extension. The 40-cycle reactions were completed in 22 min. **(B)** Lanes 1 to 4: PCR products from commercial thermal cycler. Lane 5: ladder. Lanes 6 to 9: PCR products from TTC with glass capillary tubes, using a protocol of 2 min hot-start, followed by 40 cycles of 7 s and 8 s denaturation and annealing/extension. The reactions were completed in 13 min and 3 s.

To firmly demonstrate that the TTC is as efficient as a commercial thermal cycler in amplifying nucleic acid targets, one needs to show that the TTC can achieve a very low detection limit with single digit copies of template in a single reaction. This means that the amplicons from low copy template reactions can be detected by gel electrophoresis or other means using a reasonable number of cycles (45 cycles and below). To show that the TTC not only is fast, but also extremely efficient, we used the *S*. *aureus nuc* gene primers (281 bp) and a fast PCR reaction master mix in thin-walled PCR tubes to amplify the extracted template [44]. The reactions were made with serially diluted template to allow us to prepare each reaction to contain 2, 20, 200 or 2000 copies of *S*. *aureus* genome (2 μL of template per reaction). The commercial PCR protocol needed 48 min and 18 s. The TTC condition was 2 min. hot-start at 95°C, 40 cycles of [95°C (11 s) and 60°C (17 s)] for a total of 22 min. The gel electrophoresis data (Fig 8A) shows that in 40 PCR cycles, both the TTC and the commercial thermal cycler were able to amplify enough DNA from a 20 copies/reaction sample to give a visible band in gel.

Reactions were repeated using glass capillary tubes with the same mix and concentrations. As expected, the glass capillary tubes can complete reactions with less incubation time (7 s denaturation and 8 s annealing/extension). The TTC performed highly efficient amplification in just 13 min and 3 s and the result from the gel data (Fig 8B) shows that both the commercial thermal cycler and TTC can amplify DNA from 20 copies per reaction for positive detection by gel electrophoresis. It is important to mention that both the commercial thermal cycler and the TTC did not generate enough PCR amplicons for positive gel electrophoresis identification from the tubes with a concentration of 2 copies/reaction in 40 cycles. This supports our belief that the efficiency of PCR performed by the TTC is as good as the commercial unit. The ability of this low-cost TTC in performing highly sensitive PCR amplification of reasonably long targets (~20 template copies and 281 bp) in such short time is quite remarkable.

## Conclusions

A low-cost ($130) and rapid (17 s per cycle) PCR thermal cycler was built using commercial off-the-shelf items. The use of multiple water baths, constructed with vacuum insulated stainless steel thermoses individually maintained at temperatures needed for nucleic acid denaturation and annealing/extension steps, eliminates the long ramping time needed by traditional thermoelectric-based thermal cyclers. The use of a thin layer of oil critically improves water’s ability to maintain temperatures for as long and stable as needed to perform rapid PCR without any active temperature control during the reaction. We presented data that confirms the TTC’s capability in amplifying a variety of samples by using both polypropylene plastic tubes and glass capillary tubes. This simple platform can be used in resource-limiting settings where stable or continuous power supply is not guaranteed. Unlike some rapid PCR thermal cycling approaches that are limited to amplifying short targets (<150 bp), the TTC, with limited testing, has proven that it can handle a wide range of target lengths (up to 1,522 bp have been successfully tested) and concentrations (20 copies/reaction). We also demonstrated that the TTC’s amplification efficiency matches those found in commercial cyclers since it offers the same limit of detection using the same number of cycles. The TTC has the ability to accept a wide range of PCR reaction vessels to run virtually any well-established PCR reactions without needing assay optimization. By combining our TTC and post-PCR detection schemes such as lateral-flow nucleic acid assay, we envision that low-cost rapid molecular assays can easily be developed for use in LRS.

## Supporting Information

S1 Table Set-up of Thermos water baths for denaturation and annealing/extension. (DOCX)
