# Supplementary material for: A Rapid and Low-Cost PCR Thermal Cycler for Low Resource Settings
Source: PLoS One. 2015 Jul 6;10(7):e0131701. doi: 10.1371/journal.pone.0131701 (PMC4492969; doi:10.1371/journal.pone.0131701)
Supplement: S1 Table — (DOCX) [file pone.0131701.s001.docx]

**Table S1. Set-up of Thermos water baths for denaturation and annealing/extension.**

| **Target Temperature: 55 °C** | | | |
| --- | --- | --- | --- |
| Tap water at 25 °C (mL) | Oil (mL) | Boiling water (mL) | Final temperature (°C) |
| 260 | 11 | 185 | 55.1 |
| 260 | 11 | 185 | 55.1 |
| 260 | 11 | 185 | 55.3 |
|  | | Mean final temperature (°C) | 55.17 |
|  |  | Standard deviation | 0.12 |
| **Target Temperature: 60 °C** | | | |
| Tap water at 25 °C (mL) | Oil (mL) | Boiling water (mL) | Final temperature (°C) |
| 225 | 11 | 216 | 60.6 |
| 225 | 11 | 216 | 60.7 |
| 225 | 11 | 216 | 60.8 |
|  | | Mean final temperature (°C) | 60.70 |
|  |  | Standard deviation | 0.10 |
| **Target Temperature: 63 °C** | | | |
| Tap water at 25 °C (mL) | Oil (mL) | Boiling water (mL) | Final temperature (°C) |
| 212 | 11 | 232 | 63.0 |
| 212 | 11 | 232 | 63.0 |
| 212 | 11 | 232 | 63.0 |
|  | | Mean final temperature (°C) | 63.00 |
|  |  | Standard deviation | 0.00 |
| **Target Temperature: 65 °C** | | | |
| Tap water at 25 °C (mL) | Oil (mL) | Boiling water (mL) | Final temperature (°C) |
| 200 | 11 | 245 | 65.2 |
| 200 | 11 | 245 | 64.9 |
| 200 | 11 | 245 | 65.1 |
|  | | Mean final temperature (°C) | 65.07 |
|  |  | Standard deviation | 0.15 |
| **Target Temperature: 72 °C** | | | |
| Tap water at 25 °C (mL) | Oil (mL) | Boiling water (mL) | Final temperature (°C) |
| 151 | 11 | 281 | 72.0 |
| 151 | 11 | 281 | 71.9 |
| 151 | 11 | 281 | 72.0 |
|  | | Mean final temperature (°C) | 71.97 |
|  |  | Standard deviation | 0.06 |
| **Target Temperature: ≥95 °C** | | | |
| Tap water at 25 °C (mL) | Oil (mL) | Boiling water (mL) | Final temperature (°C) |
| 0 | 11 | 418 | 96.7 |
| 0 | 11 | 448 | 97.3 |
| 0 | 11 | 431 | 97.1 |
|  | | Mean final temperature (°C) | 97.03 |
|  |  | Standard deviation | 0.31 |

Each Thermos water bath was set up independently for 3 times, and the final temperature was measured for each to confirm the reproducibility of the set-up.
